# The effects of human Wharton’s jelly cell transplantation on the intervertebral disc in a canine disc degeneration model

**DOI:** 10.1186/s13287-015-0132-z

**Published:** 2015-08-27

**Authors:** Yan Zhang, Hui Tao, Tao Gu, Mingyue Zhou, Zhiwei Jia, Gangqiang Jiang, Chun Chen, Zhihua Han, Cheng Xu, Deli Wang, Qing He, Dike Ruan

**Affiliations:** Department of Orthopedic Surgery, Navy General Hospital, No. 6 Fu-cheng Road, Beijing, 100048 P R. China; VIP Neurology Department, Navy General Hospital, No. 6 Fu-cheng Road, Beijing, 100048 P R. China; Department of Spine Surgery, The First Affiliated Hospital of Anhui Medical University, No. 218 Jixi Road, Hefei, Anhui China

## Abstract

**Introduction:**

Cell-based therapy was a promising treatment method for disc degenerative diseases. Wharton’s jelly cell (WJC) has been explored to cure various human diseases, while it still remains unknown about this MSC for disc repair. In our prior work, WJCs could differentiate into nucleus pulposus (NP)-like cells by co-culturing with NP cells in vitro. Thence, the aim of this study was further to investigate the survival and function of WJCs in vivo after transplantation into degenerated canine discs.

**Method:**

WJCs were isolated from human umbilical cords and labeled with EGFP. The degeneration of L4-5, L5-6, and L6-7 discs of beagles was induced by aspirating the NP tissues. Four weeks after the operation, the injured discs were left to be no treatment at L4-5 (DS group), injected with 0.9 % saline at L5-6 (FS group), and transplanted with EGFP-labeled WJCs at L6-7 (TS group). In all animals, the intact disc L3-4 served as a control (CS group). The animals were followed up for 24 weeks after initial operation. Spine imaging was evaluated at 4, 8, 12, and 24 weeks, respectively. Histologic, biomechanics and gene expression analyses were performed at 24 weeks. Immunohistochemistry for aggrecan, types II collagen, SOX-9 was employed to investigate the matrix formation in the NP.

**Results:**

The TS group showed a significantly smaller reduction in the disc height and T2-weighted signal intensity, and a better spinal segmental stability than DS and FS groups. Histologic assay demonstrated that WJCs were specifically detected in TS group at 24 weeks and the discs of TS group maintained a relatively well preserved structure as compared to the discs of DS and FS groups. Furthermore, real-time PCR and immunohistochemistry demonstrated that expressions of disc matrix genes, aggrecan, type II collagen, and SOX-9, were up-regulated in TS group compared to DS and FS groups.

**Conclusion:**

WJCs could not only survive in the degenerate IVDs, but also promote the disc matrix formation of aggrecan and type II collagen in the degenerate IVDs. It may have value in cell-based therapy for degenerative disc disease.

## Introduction

Chronic low back pain (LBP) is one of the most common musculoskeletal disorders in humans. Intervertebral disc (IVD) degeneration and associated pathology have been implicated as a major cause of LBP [1]. Studies have revealed that the nucleus pulposus (NP) of the IVD played a prominent role in both the onset and progression of IVD degeneration, which was characterized by the loss of cells in NP and followed by decreased function of producing extracellular matrix (ECM) [2–4]. Current treatment for degenerative disc disease is usually limited to conservative and invasive care, including nonsurgical modalities and operative methods. None of these treatment strategies produce reliable outcomes, however, because their aim was only to relieve acute symptoms and they failed to promote tissue regeneration or halt the process of IVD degeneration. In response to this challenge, it is of great significance to develop novel technologies to manage IVD degeneration. Cell-based therapy has emerged as a promising treatment method for degenerative disc disease.

The lack of ideal exogenous cells, however, remains a serious problem among the methods. In recent years, many cell sources such as autologous NP cells have been evaluated to promote IVD tissue regeneration [5]. But human NP tissues contain few healthy autologous cells except a small quantity of NP progenitor cells [6]. Alternatively, mesenchymal stem cells (MSCs) have been explored as a promising cell source for repairing degenerate IVD [7–9]. MSCs are multipotent stem cells that can be isolated, expanded, and stimulated to differentiate into a variety of cells, including osteoblasts, chondrocytes, myocytes, adipocytes, and beta pancreatic islet cells [10, 11]. Studies have shown that MSCs could be induced to differentiate into an NP-like phenotype when stimulated appropriately [12–15]. MSCs can also be injected directly, or together with a scaffold, into the degenerate IVD, where they can differentiate into disc cells, produce ECM, and reestablish healthy disc function [16, 17]. Taking these results together, MSCs have been highlighted as a potential therapeutic option for the IVD regenerative medicine.

MSCs are widely distributed in a variety of human tissues such as fetal liver, umbilical cord, bone marrow, adipose tissue, joint synovia, muscle, and dermis. Although bone marrow represents the main source of MSCs for both experimental and clinical studies, the number and the proliferative/differentiation capacity of bone marrow MSCs (BMSCs) significantly decrease with age [18, 19]. Thus, it is necessary to look for possible alternative sources of MSCs. Human umbilical cord Wharton’s jelly tissue is one such alternative and contains stem cells similar to adult MSCs [20, 21]. These cells, known as Wharton’s jelly cells (WJCs), could self-renew and differentiate into various cell types [22], such as cardiomyocytogenic cells, muscle cells [23], osteogenic cells [24, 25], adipogenic cells [26], and neural cells [27–29]. WJCs also have been explored to cure various human diseases [30, 31] such as cancer [32], liver disease [33], cartilage injuries [34], cardiovascular disease [35], skin wounds [36], osteoarthritis [37], obesity [38], and diabetes [39]. Furthermore, WJCs have also been proposed as a potential application in connective tissue repair [40] and even degenerative disc disease. One study has demonstrated that WJCs could differentiate into cells with an immature NP-like phenotype under suitable environmental conditions [41]. Our earlier study also found that WJCs could differentiate into NP-like cells by coculturing with the NP cells and induce the disc matrix formation [42]. Furthermore, in a direct cell-to-cell contact condition, WJCs could upregulate the expression of the disc matrix aggrecan, type II collagen, and SRY-box 9 (SOX-9), suggesting that the direct cell–cell contact was important for WJC differentiation. Above all, these studies propose that WJCs might be promising seed cells in cell-based treatment for degenerative disc disease. This study was therefore conducted to explore the potential of WJC implantation to restore degenerate IVD. We used imaging and histological analyses to investigate survival and regenerative effects of WJCs after transplantation into the degenerate IVDs in a canine disc degeneration model.

## Materials and methods

### Wharton’s jelly cell isolation, culture, and flow cytometry analysis

This study was approved by the Navy General Hospital Ethical Committee. With the consent of the parents, human umbilical cord Wharton’s jelly was collected from infants delivered by full-term normal labor. The cells from the Wharton's jelly tissue were isolated as described previously [26]. Briefly, after removal of blood vessels and epithelium, the Wharton’s jelly was cut into pieces about 1.5–2.5 mm^3^, and digested for 18 hours at 37 °C with 0.2 mg/ml collagenase (Sigma St. Louis, Missouri, USA) solution in serum-free medium containing 100 U/ml penicillin, 100 mg/ml streptomycin, and 2.5 mg/ml amphotericin B. The isolated cells were suspended in Dulbecco's modified Eagle’s medium (DMEM)/F12 nutrient mixture containing 20 % fetal bovine serum (FBS). When WJCs were cultured to reach 80–90 % confluence, they were passaged and seeded at a density of 4 × 10^3^/cm^2^ in DMEM/F12 with 10 % FBS. WJCs at passage 3 were taken for flow cytometry analysis as described previously [42]. The antibodies used were phycoerythrin (PE)-conjugated CD105, CD73, CD45, CD29, CD166, human leukocyte antigen (HLA)-DR, HLA-ABC, and fluorescein isothiocyanate (FITC)-conjugated CD34 and CD90 (all eBioscience, Santiago, California, USA).

### Transfection of Wharton’s jelly cells with AAV2-EGFP viral vector

To facilitate analysis of the survival of transplanted WJCs in the NP region, the WJCs were infected with AAV2-EGFP, an adeno-associated virus vector expressing the enhanced green fluorescent protein (EGFP) gene. Briefly, after the third passage, the cells were cultured in 25 cm culture flasks until reaching 80 % confluence. The cells were then washed with phosphate-buffered saline (PBS; Gibco Grand Island, New York, USA) and infected with AAV2-EGFP at multiplicity of infection 10^5^ vector genome/cell. Noninfected cells served as a negative control. The cells were incubated for 1 hour, washed in PBS, and cultured in DMEM/F12 with 10 % FBS. After 5 days, green fluorescent protein (GFP) expression was observed using a fluorescence microscope, and vector incorporation was tested by flow cytometry.

### Disc degeneration model and transplantation of Wharton’s jelly cells

Eighteen skeletal mature beagles (11–13 months old), weighing approximately 10 kg, were used in this study. Animal experiments were approved by Navy General Hospital Animal Experimentation Committee. All animals were healthy and free of infection, and were given X-ray and magnetic resonance imaging (MRI) scans of the spine to assure the absence of IVD degeneration-related diseases before the study. Surgical procedures were performed under general anesthesia by intramuscular administration of ketamine hydrochloride injection (0.1 ml/kg) and xylazine hydrochloride (0.08 ml/kg). The anterior surfaces of three consecutive lumbar discs (L4–5, L5–6, and L6–7) were exposed through an anterior–lateral approach. To induce disc degeneration, an 18-gauge needle was inserted at the center of the disc through the annulus fibrosus (AF) into the NP. The NP was then aspirated using a 10 ml syringe, as described previously [43, 44]. The mean weight of the NP aspirated from a single disc was 14.5 ± 2.7 mg. Four weeks after the operation, the injured disc L4–5 remained with no treatment as a degenerative segment (DS group), the injured disc L5–6 was injected with 100 μl of 0.9 % saline (FS group), and the injured disc L6–7 was transplanted with 100 μl of 1 × 10^6^ EGFP-labeled WJCs (TS group). In all animals, the intact L3–4 disc served as a noninjured control (CS group). The animals were followed up for 24 weeks after the initial operation. The changes in the lumbar discs were assessed using radiographs, MRI, biomechanical testing, (immuno)histology, and gene expression analysis.

### Radiological analysis of disc height

Lateral radiographs were taken at 0, 4, 8, 12, and 24 weeks. A fluoroscopic imaging intensifier (radiographs; 68 kV, 10 mA, 100 cm distance) was used. The digitized radiographic images were stored and evaluated using the CDmanager software program (version 6.5.0.0; Silver MedInfo. Tech., Beijing, China). The measurements included vertebral body height and IVD height. The disc height index (DHI) at each level was determined based on the method of Lu et al. [45]. Changes in the DHI were expressed as %DHI and normalized to the measured preoperative IVD height:

$$ \%\mathrm{D}\mathrm{H}\mathrm{I}=\left(\left(\mathrm{postoperative}\ \mathrm{D}\mathrm{H}\mathrm{I}/\mathrm{preoperative}\ \mathrm{D}\mathrm{H}\mathrm{I}\right)\times 100\right). $$

### Magnetic resonance imaging

MRI scans were taken to evaluate signal changes in T2-weighted images at 0, 4, 8, 12, and 24 weeks after the first operation in all groups. MRI scans were obtained in T2-weighted images in all groups at each time point in the sagittal and axial planes using a 1.5 T scanner (Magneton 63P/4000; Siemens, Iselin, NJ, USA). T2-weighted sections in the sagittal plane were obtained under the following settings: fast spin echo sequence with time to repetition of 2500 seconds and time to echo of 85 seconds; 384 (h) × 224 (v) matrix; field of view of 260; and four excitations. The section thickness was 3 mm with a 0.3 mm gap. The digitized MRI scans were stored on computer, and the CDmanager software program was used to determine the grayscale of the NP and the cerebrospinal fluid at the same level on a T2-weighted sagittal MRI scan. The disc relative gray index (RGI) was calculated by dividing the gray value of the NP by the gray value of the cerebrospinal fluid. The MRI scans were also evaluated using the Pfirrmann classification before and 24 weeks after the first operation [46–48]. Three observers, blinded to this study, performed the measurements and grading for radiological and MRI scans. The means of these measurements were taken, and the average grade of the three observers was used as the final grade for each disc. The intraobserver reliability based on readings at two time intervals 1 month apart was κ = 0.92, showing an excellent agreement.

### Biomechanical analysis

At 24 weeks, all beagles were killed with an excess dose of ketamine hydrochloride and xylazine hydrochloride injection, and the 18 spines were harvested. Biomechanical testing was performed on the spinal motion segment with all ligamentous attachments and all of the muscles removed. The spinal column was embedded in denture base resin, with the L3–7 vertebral body remaining outside. Every vertebra of L3–7 was marked by markers in the same position (the specific position of every vertebra), and two video cameras recorded the motion of the markers from different direction when the biomechanical test was being carried out. The biomechanical analysis was performed using a biomechanical machine (MTS 858 Mini Bionix II; Minneapolis, Minnesota, USA). The fresh spinal specimens were kept wet during the process of testing. Before carrying out the test, a preliminary load was stressed on the specimen to reduce the interference from the viscoelasticity of the specimen. The pre experiment was repeated five times (30 seconds each time) with 1 N preloading. To determine the multidirectional flexibility properties, the multidirectional unconstrained bending moments (flexion and extension (±3 N m*, x* axis), left and right torsion (±3 N m, *y* axis), and left and right bending (±3 N m, *z* axis)) were applied to the superior end of the vertically oriented specimen, while the caudal portion of the specimen remained fixed to a testing platform. As the sample was loaded, the motions of all markers were recorded by two video cameras. The multidirectional range of motion (ROM) of individual motion segments of every IVD was measured according to the motions of markers. The ROM was measured five times respectively and the mean ROM was used for the statistical analysis.

### Macroscopic observations and assessment of survival transplanted Wharton’s jelly cells

After the biomechanical testing, the L3–4/L4–5/L5–6/L6–7 discs were isolated intact, preserving the bilateral cartilage endplate and some vertebral body. The discs from one dog were cut coronally at the center of the disc for macroscopic evaluation. The discs from another dog were used to identify the surviving injected cells in the NP tissues. The NP tissue was separated from the disc and was frozen in tissue-freezing medium (Fisher Scientific, Pittsburgh, PA, USA) and sectioned 60 μm thick in a transverse plane from each disc. The GFP-positive cells were observed under fluorescence microscopy using a Texas Red filter (Chroma, Rockingham, VT, USA).

### Histological and immunohistochemical analysis

The remaining discs of 16 dogs were cut transversally at the center of the NP. One half of every disc was used for histological studies, and the other half was used for real-time PCR analysis of gene expression.

The NP tissues were isolated immediately and were fixed in 10 % neutral-buffered formalin for 72 hours and processed for paraffin embedding and cut into transversal sections (6 μm thick) using a microtome. The sections were stained with hematoxylin and eosin for evaluation.

Immunohistochemical detection of GFP, type II collagen, aggrecan, and SOX-9 was performed using formalin-fixed sections obtained as described above. Briefly, NP tissue sections were maintained at room temperature for 60 minutes and dewaxed by xylene twice (10 minutes each time). The tissues were then rehydrated by a series of 5-minute washed in 100 %, 95 %, 80 %, and 70 % ethanol, followed by 5-minute washed in distilled water and three consecutive 3-minute washed with PBS. After inactivating the endogenous peroxidase by incubating in 3 % hydrogen peroxide for 10 minutes, antigen retrieval was performed by heating the samples at 95 °C for 20 minutes in 10 mM sodium citrate (pH 6.0). After nonspecific binding was blocked by incubating with 10 % normal goat serum (Solarbio Company, Beijing, China) for 20 minutes, the NP tissue sections were labeled overnight at 4 °C with primary antibody: anti-GFP (1:100 dilution), anti-type II collagen (1:200 dilution), anti-aggrecan (1:100 dilution), and anti-SOX-9 (1:50 dilution) polyclonal antibodies (immunoglobulin G) (Santa Cruz Biotech, Santa Cruz, CA, USA). These primary antibodies were not specific for human, and could also be reactive with additional species including canine and porcine. The NP sections were then incubated for 60 minutes each with a horseradish peroxidase-labeled secondary antibody and then streptavidin–peroxidase (Santa Cruz Biotech). After washing with PBS, the sections were incubated with 3,30-diaminobenzidine substrate until a brown color developed. Finally, the sections were counterstained with hematoxylin. The slides were then dehydrated by a series of 2-minute washes in 50 %, 70 %, 95 %, 95 %, and 100 % ethanol. After two consequent 2-minute washes with xylene, the slides were sealed with coverslips. Cartilage of the femoral head of the beagle served as a positive control, and the negative control included the use of PBS instead of primary antibody, with all other conditions kept the same. To quantify the immunohistochemical results, staining intensity was analyzed using the Image-Pro Plus 6.0 (Media Cybernetics, Rockville, Maryland, USA). The area of interest in all sections was analyzed, and the mean density was calculated by integrated optical density divided by the area.

### Real-time PCR analysis of gene expression

Total RNA was extracted from the NP using the Trizol reagent (Invitrogen, Carlsbad, CA, USA) according to the manufacturer’s instructions. RNA was reverse transcribed into cDNA using AMV reverse transcriptase (Takara Biotechnology, Dalian, China). After the cDNA had been obtained by reverse transcription, relative gene expressions of aggrecan, type I collagen, type II collagen, and SOX-9 were determined by real-time PCR and normalized to the glyceraldehyde-3-phosphate dehydrogenase housekeeping gene. These primers were designed using Primer Premier 6.0 software (PREMIER Biosoft, palo alto, California, USA) (Table 1). The Mini Opticon™ Detector System (Bio-Rad, Hercules, California, USA) and the SYBR Green PCR kit (Takara Biotechnology, Dalian, China) were used for real-time PCR analysis. The real-time PCR consisted of an initial enzyme activation step at 95 °C for 20 seconds, followed by 40 cycles of 95 °C for 5 seconds and 60 °C for 20 seconds. A cycle threshold (Ct) value was obtained for each sample, and triplicate sample values were averaged. The 2^–ΔΔCt^ value was then used to calculate relative expression of each target gene [49]. The data presented (mean) were from three independent experiments in which both sample sets were analyzed in triplicate.

**Table 1:** Primers designed using Primer Premier 6.0 software

Gene	Gene bank	Sequence
Type II collagen	NM_001006951.1	5′-GAAGAGCGGAGACTACTGGATTG-3′
		5′-AGGCGGAGGAAGGTCATCTG-3′
Type I collagen	NM_001003090.1	5′-TTCTGGTCCTCGTGGTCTCC-3′
		5′-CTTCACCGTCATCTCCGTTCTT-3′
Aggrecan	NM_001113455.1	5′-GCAGGACCAGACTGTCAGATAC-3′
		5′-TCCAGGCGTGTGATGAAGAAC-3′
SOX-9	NM_001002978.1	5′-CCAGCGAACGCACATCAAGA-3′
		5′-TGTAGGTGAAGGTGGAGTAGAGG-3′
GAPDH	NM_001003142.1	5′-TCTGCTCCTTCTGCTGAT-3′
		5′-GCCTGCTTCACTACCTTC-3′

Primer Premier 6.0 software from PREMIER Biosoft (palo alto, California, USA)

*GAPDH* glyceraldehyde-3-phosphate dehydrogenase, *SOX-9* SRY-box 9

### Statistical analysis

SPSS version 15.0 software (Chicago, Illinois, USA) was used for statistical analysis. The comparison of group means between the CS group, the TS group, the FS group, and the DS group was determined using repeated-measure analysis of variance and Fisher’s least significant difference post-hoc test, in which *p* <0.05 was considered significant. The Mann–Whitney *U* test was used to analyze the nonparametric data from MRI Pfirrmann grading.

## Results

### Characterization of Wharton’s jelly cells and expression of cell surface receptors and green fluorescent protein in WJCs

**Fig. 1:**
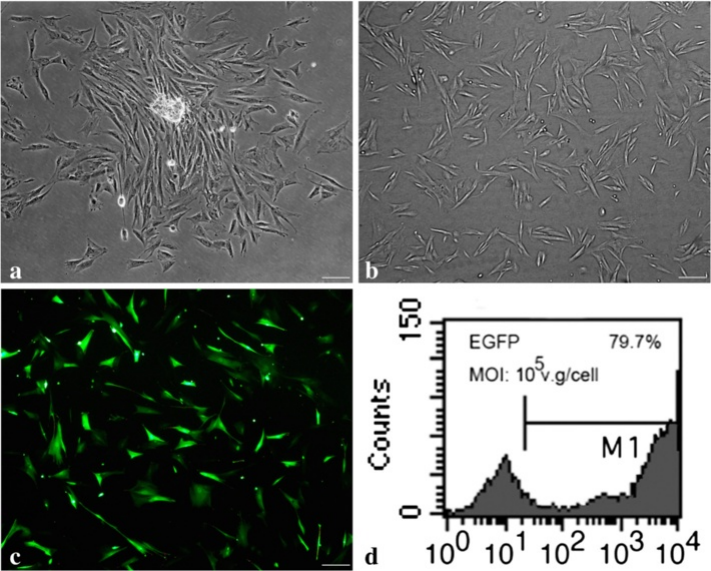
Morphology of human WJCs and expression of GFP in WJCs. **a** After initial culturing for 3 days, fibroblast-like cells migrated out from the enzyme-digested Wharton’s jelly tissues and adhered to the culture dish, and the fibroblastic colony formation could be observed. **b** After 3 days in culture, passage 3 Wharton’s jelly MSCs appeared to be mostly spindle-shaped or triangular. **c** Fluorescent imaging of WJCs infected with AAV2 expressing EGFP with multiplicity of infection (*MOI*) of 1 × 10^5^ vector genome/cell. **d** Vector incorporation was 79.7 % at 5 days after AAV2-EGFP infection (bar = 50 μm). *EGFP* enhanced green fluorescent protein

The primary WJCs in vitro displayed adherent growth and could form cell colonies (Fig. 1a). The subcultured WJCs exhibited fibroblast-like morphology and uniform distribution in the tissue culture plastic surface (Fig. 1b). Flow cytometric analysis showed that the cells expressed CD90, CD73, CD105, CD29, and HAL-ABC, but were negative for CD34, CD45, and HLA-DR (Table 2). GFP expression was confirmed using a fluorescence microscope 5 days after AAV2-EGFP infection (Fig. 1c), and vector incorporation was 79.7 % using flow cytometric analysis (Fig. 1d).

**Table 2:** Human Wharton’s jelly cells of passage 3 express cell surface markers typical to mesenchymal stem cells

Target	% positive cells^a^	MFI
CD90	99.7	430
CD73	99.1	27
CD105	99.2	98
CD29	99.6	251
CD166	99.1	19
HLA-ABC	98.6	10
CD34	0.2	12
CD45	0.6	31
HLA-DR	0.5	15

Values represent a mean of triple samples (*n* = 3). Data obtained via flow cytometry analysis

^a^Percentage of total number of cells expressing the markers

*HLA* human leukocyte antigen, *MFI* mean fluorescence intensity

### Changes in the intervertebral disc height

**Fig. 2:**
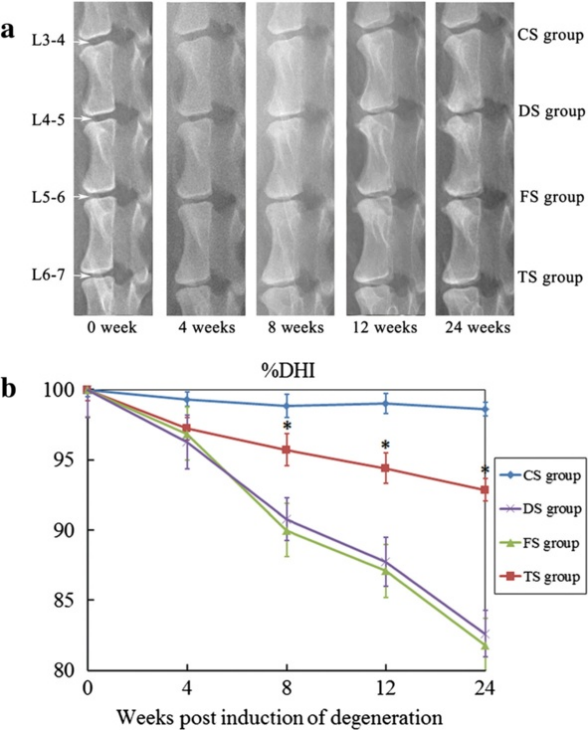
Radiographic assessment. **a** Representative radiographic images from the WJC transplanted group (TS group), degenerated control groups (FS and DS groups), and intact disc group (CS group) at 0, 4, 8, 12, and 24 weeks after the first operation. **b** %DHI was measured at each time point to quantify changes in disc height. Note: %DHI in the TS group was significantly higher than those of the discs in the FS and DS groups at 8, 12, 18, and 24 weeks after the first operation. Each data point represents the mean and standard deviation of 18 samples (*p <0.05, *n* = 18). *%DHI* changes in the disc height index

We first evaluated the effect of WJC transplantation on the disc height. At 4 weeks after the initial operation, narrowing of the disc space was observed in all three operated groups (DS, FS, and TS groups). The %DHI of the DS and FS groups continued to decrease in the entire experimental period. The %DHI of the TS group also decreased gradually over time but was higher than that of the DS and FS groups at 8, 12, and 24 weeks (*p* <0.05) (Fig. 2).

### Magnetic resonance imaging assessment

**Fig. 3:**
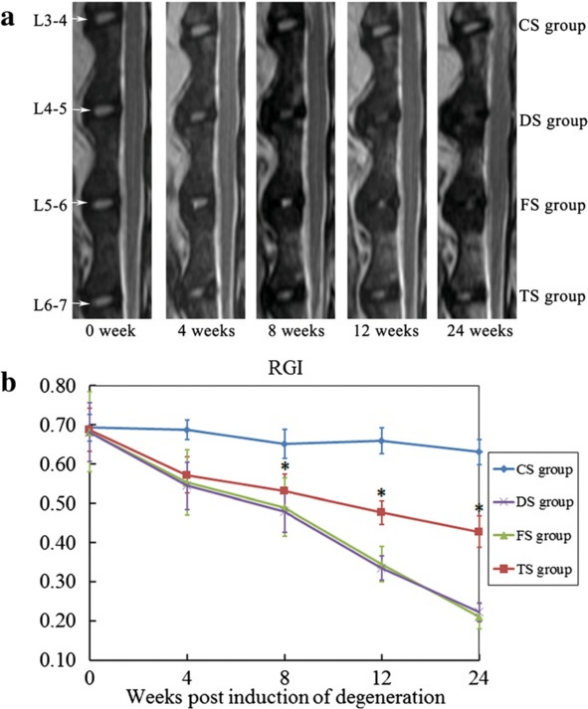
MRI analysis. **a** Representative MRI scans of the discs from the WJC transplanted group (TS group), degenerated control groups (FS and DS groups), and intact disc group (CS group) at 0, 4, 8, 12, and 24 weeks after the first operation. **b** Disc relative gray index (*RGI*) at 0, 4, 8, 12, 18, and 24 weeks after the first operation. Note: RGI in the WJC transplanted discs (TS group) was significantly higher than those of the discs in the FS and DS groups at 8, 12, 18, and 24 weeks after the first operation. Each data point represents the mean and standard deviation of 18 samples (*p < 0.05, *n* = 18)

**Fig. 4:**
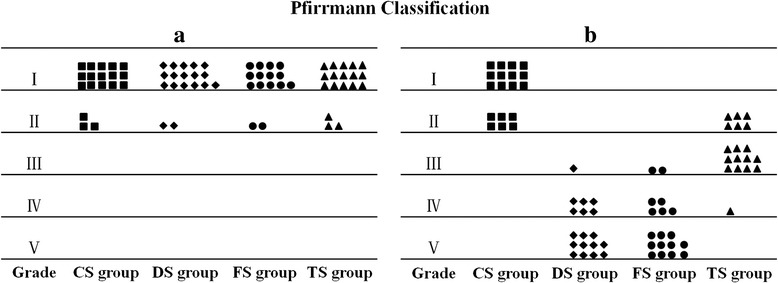
Analysis of signal changes in T2-weighted images using the Pfirrmann classification. **a** Discs showed no degeneration in all groups before the first operation. **b** There was significantly lower grading of MRI scans in the TS group compared with DS and FS groups at 24 weeks after the first operation (*p* <0.01)

As shown in Fig. 3, the mean RGI of the CS group barely changed throughout the study. The mean RGI in the DS, FS, and TS groups showed a significant decrease after the first operation compared with the CS group (*p* <0.05). The discs in the TS group showed stronger signal intensity than those in the DS and FS groups at 8 weeks, and this result was sustained up to 24 weeks (*p* <0.05). No significant differences were seen among the DS and FS groups. The Pfirrmann classification analysis did not show disc degeneration in all groups before the first operation (Fig. 4a). The Pfirrmann classification in the TS group was significantly higher compared with the CS group (*p* <0.01), but significantly lower compared with the DS and FS groups at 24 weeks (*p* <0.01) (Fig. 4b). Together, these results indicated that the NP signal intensity considerably decreased at 4 weeks after the second operation, but the WJC transplantation reduced the decrease of the NP signal intensity over the entire experimental period.

### Results of biomechanical analysis

**Fig. 5:**
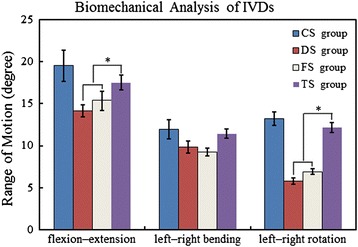
Analysis of ROM of the discs. ROM of left–right bending was not statistically different in the four groups. However, the ROM of flexion–extension and left–right rotation in the TS group was larger than that of the DS and FS groups at 24 weeks. *Significant difference between the TS group and the DS or FS group, *n* = 18. *IVD* intervertebral disc

We further compared the biomechanical effect from the three operated discs (DS, FS, and TS groups) and intact disc (CS group). As shown in Fig. 5, the ROM of flexion–extension and left–right rotation significantly decreased in the DS or FS groups compared with the CS group. The ROM of flexion–extension and left–right rotation in the TS group showed significantly less reduction compared with the DS or FS groups. The ROM of left–right bending, however, was not statistically different between the four groups. The result showed that transplantation of the WJCs into the IVD maintained the ROM of flexion–extension and left–right rotation.

### Macroscopic findings

**Fig. 6:**
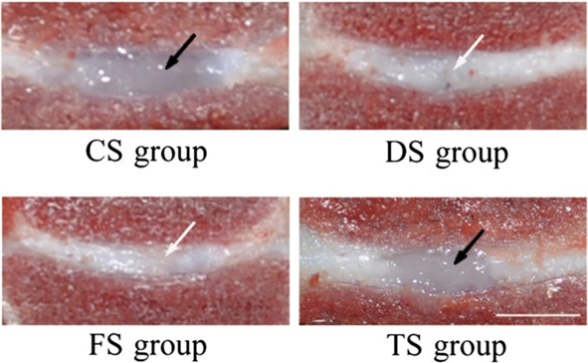
Typical macroscopic views of IVDs from the CS, DS, FS, and TS groups. Disc in the TS group shows an oval-shaped gel-like NP structure similar to that of a disc from the CS group (*black arrow*). Discs from the DS and FS groups show loss of the normal NP structure (*white arrow*) (bar = 5 mm)

As shown in Fig. 6, the macroscopic evaluation showed that the NP in the CS group was hyaline and gelatinous semi-fluid, and the NP in the TS group maintained a relatively normal oval-shaped structure similar to that in the CS group at 24 weeks. The DS and FS groups showed disc space narrowing and dense scar tissues in the NP. The discs of the TS group maintained the disc structure better than that of the DS and FS groups. However, the figure showed the gross appearance of the IVDs from one animal only.

### Survival of transplanted Wharton’s jelly cells in the intervertebral discs

**Fig. 7:**
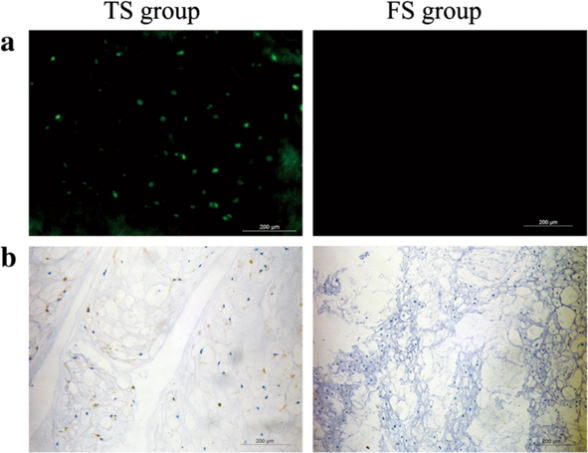
Survival of the EGFP-labeled WJCs in the transplanted and control discs. **a** Fluorescence microscopy demonstrated the survival of the EGFP-labeled WJCs in the TS group disc. **b** Immunohistochemical micrographs illustrate the survival of the EGFP-labeled WJCs in the TS group disc. Note: no positive cell was observed in the FS group disc

In this study, EGFP-labeled WJCs were transplanted into the degenerate IVD in the TS group. One animal’s discs were used to detect the survival of transplanted WJCs by fluorescence microscopy. The other animals’ discs were analyzed by immunohistochemistry. As shown in Fig. 7, the fluorescence microscopy and immunohistochemical micrographs confirmed the survival of the EGFP-labeled WJCs in the discs of the TS group. No GFP-positive cells were observed in the discs of the FS group.

### Result of histological and immunohistochemical analysis

**Fig. 8:**
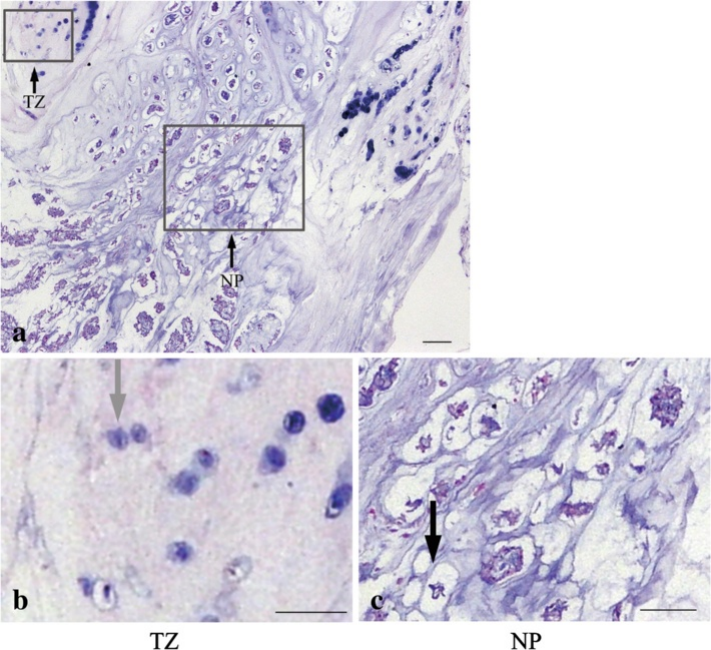
Histological image of the disc in the CS group. **a** Hematoxylin and eosin stain showed that the disc contains the nucleus pulposus (*NP*) and transitional zone (*TZ*). **b** The TZ includes cartilage cells in the sections indicated by the *gray arrow*. **c** The NP includes vacuolar degeneration notochordal cells in the sections indicated by the *black arrow*. Bar = 100 μm

**Fig. 9:**
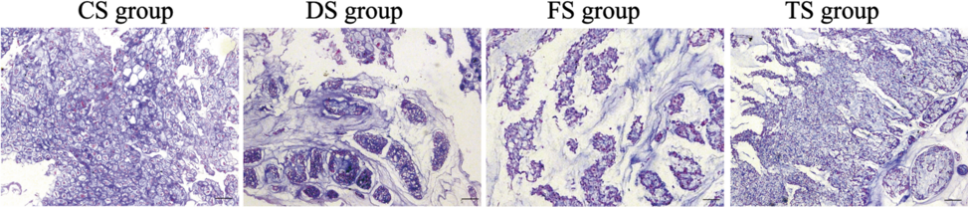
Typical histological images of the NP in four groups. The paraffin sections of the NP tissues were stained by hematoxylin and eosin. These micrographs illustrate the typical images of the NP sections from the WJC transplanted group (TS group), degenerated control groups (DS and FS groups), and intact NP group (CS group) (bar = 100 μm)

**Fig. 10:**
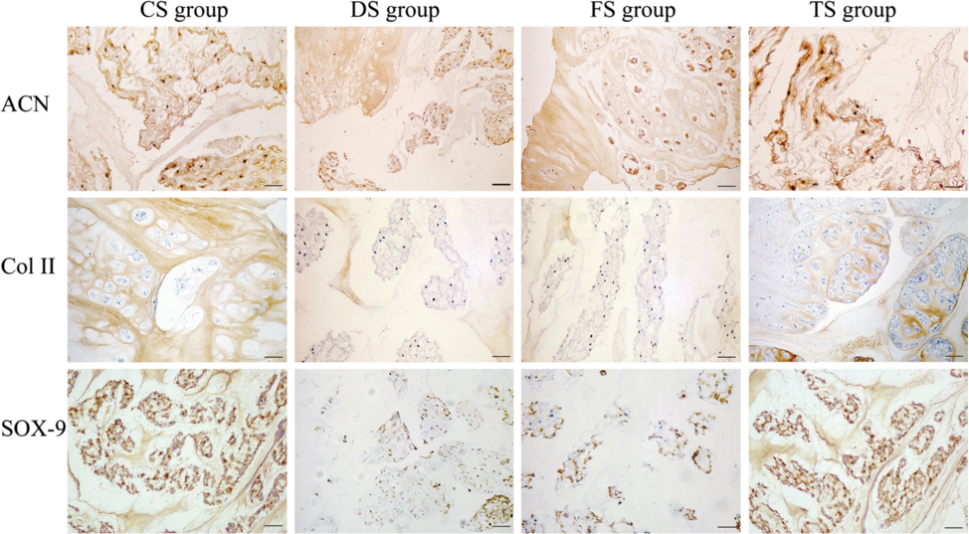
Typical immunohistochemical images. The immunohistochemical micrographs illustrate the typical images of the NP sections which were stained by an antibody against SRY-box 9 (*SOX-9*), aggrecan (*ACN*), and type II collagen (*Col II*). Staining of the NP for SOX-9, aggrecan, and type II collagen for the CS and TS groups was stronger than that for the DS and FS groups

The beagle is a chondrodystrophic dog, and the IVD degeneration occurs earlier and faster than that in nonchondrodystrophic dogs. As shown in Figs. 8 and 9, early degenerative changes of the IVD were detectable in the histologic results which showed vacuolar degeneration in all groups. The degenerative changes of the NP in the DS and FS groups were more apparent than those in the CS and TS groups. To further demonstrate the effect of WJC transplantation on the disc matrix, the NP sections were stained by SOX-9, type II collagen, and aggrecan antibodies. As shown in Fig. 10, the immunohistochemical staining indicated that the NP in the TS and CS groups was strongly positive for SOX-9, type II collagen, and aggrecan. The staining intensity decreased or did not show significant intensity in the NP of the DS and FS groups. Immunohistochemical staining intensity analysis using Image-Pro Plus 6.0 showed that the staining intensity of aggrecan, type II collagen, and SOX-9 was significantly higher in the TS group than that in the DS and FS groups (*p* <0.05).

### Result of gene expression analysis

**Fig. 11:**
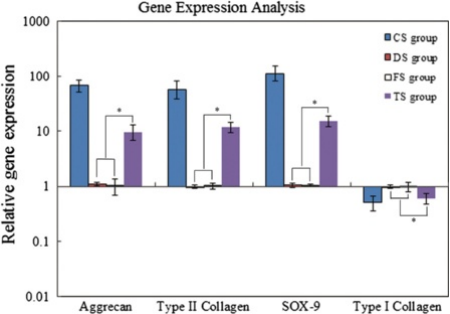
Real-time PCR analysis. Real-time PCR was used to analyze the levels of the disc matrix components aggrecan, type II collagen, type I collagen, and SOX-9 mRNAs in the disc tissues from the CS, DS, FS, and TS groups. Expression was normalized to the average of the housekeeping gene (glyceraldehyde-3-phosphate dehydrogenase) and the DS group. The results showed that aggrecan, type II collagen, and SOX-9 mRNA expression markedly increased in discs from the TS group compared with the DS and FS groups. *Statistical significance (*p* <0.05) from the DS and FS groups. *SOX-9* SRY-box 9

To confirm the effect of WJC transplantation on the disc matrix, real-time PCR was used to measure the levels of aggrecan, type II collagen, type I collagen, and SOX-9 in the CS, DS, FS, and TS groups. As shown in Fig. 11, the levels of aggrecan, type II collagen, and SOX-9 mRNAs were decreased in the DS, FS, and TS groups as compared with the CS group, while the type I collagen expression was significantly increased in the DS, FS, and TS groups (*p* <0.05). Importantly, we found that the levels of aggrecan, type II collagen, and SOX-9 mRNAs were significantly higher in the TS group than those in the DS and FS groups, and the type I collagen expression was significantly lower in the TS group than that in the DS and FS groups (*p* <0.05). Together, the results show that WJC transplantation increased the expression of aggrecan, type II collagen, and SOX-9.

## Discussion

A number of reports have shown that transplantation of adult MSCs including BMSCs [44], adipose-derived stem cells (ADSCs) [50], synovial MSCs [51], and umbilical cord blood MSCs [52] could repair the degenerate IVD in vivo, but the effect and role of WJCs in the degenerative IVDs in vivo have not been explored. By tracking the fate of EGFP-tagged WJCs and observing the role of WJCs in the degenerative IVDs in vivo, we firstly showed that WJC transplantation could survive up to 20 weeks in the degenerate IVDs, and further demonstrated that WJCs could decelerate the progressive degeneration of IVD in a canine degeneration model.

The imaging outcomes and Pfirrmann classification were two major parameters for evaluating IVD degeneration in the clinical. After WJC transplantation, we found that the decline of %DHI in the TS group was decreased compared with the DS and FS groups at the beginning of 4 weeks. On T2-weighted imaging, the NP in the TS group showed higher RGI than that in the DS and FS groups. We also used Pfirrmann classification to assess the signal intensity of T2-weighted images on MRI. The TS group showed a significantly lower Pfirrmann grading compared with the DS and FS groups at 24 weeks, and the results were consistent with that of RGI. This observation was consistent with Hiyama et al.’s study [44]. They showed that the disc regeneration was identified on radiological and MRI scans after transplantation at 4 weeks after induction of degeneration. However, in the study by Chun et al. [50] there were no signal changes in the disc space after transplantation of ADSCs. The reason for this might be that the researchers transplanted the ADSCs at 19 weeks after induction of degeneration. The degeneration of discs was so severe that few ADSCs survived in the discs, and the regeneration might not be apparent by MRI. We therefore speculate that the cell transplantation treatment might be more effective in the early period of IVD degeneration.

To determine whether xenogeneic WJCs could survive in the IVDs, the EGFP-labeled WJCs were transplanted into canine degenerate IVDs. At 20 weeks after transplantation, the viable EGFP-labeled WJCs could be detected in the IVDs of one animal by fluorescence microscopy and in all IVDs of the TS group by immunohistochemistry. This suggested that WJCs could survive up to 20 weeks in the degenerate IVDs. Similar research findings have also been obtained in porcine [53], rabbit [50], and rat [54] models using xenogeneic BMSCs, synovial MSCs, and ADSCs. In the study by Henrikkson et al. [53], MSCs were detected by immunohistochemical staining of mouse antihuman nuclei (HNA) at 6 months after transplantation to the porcine discs and were confirmed to differentiate toward chondrocyte-like cells by detecting human specific collagen II, aggrecan, and SOX9 using human TaqMan Gene Expression Assays. In the present study, we used EGFP for tracking of transplanted WJCs that were also used in the study by Miyamoto et al. [51]. In this study, human WJCs could survive at least 20 weeks in a canine model of IVD degeneration. We believe there are two reasons for this survival: WJCs are immunologically privileged and can be used for allogenic and xenogeneic transplantation; and the NP is absent of vasculature and cannot generate an immune response. So the “immunologically privileged” environment of the avascular NP might account for long-term viability of xenogeneic stem cells.

Different from the research by Henrikkson et al. [53], the primers used in real-time PCR and the primary antibodies used in immunohistochemistry were not canine or human specific in our study. The real-time PCR and immunohistochemistry results demonstrated that WJC transplantation significantly increased the expression of disc ECM components such as aggrecan and type II collagen compared with the DS and FS groups. These results suggested that the transplanted WJCs could establish a matrix-producing function in the degenerate IVDs. This was in keeping with many previous studies [16, 50, 55–58] which showed that transplantation of MSCs seems to exert corrective, therapeutic effects on the degenerate IVD even though there was no sufficient evidence to confirm the transplanted WJCs differentiating into NP cells. Furthermore, our previous studies demonstrated that the WJCs could differentiate into NP-like cells by coculturing with the disc NP cells in vitro [42]. Therefore, it could be deduced that the transplanted WJCs and their progeny perhaps differentiated into NP-like cells in the stromal microenvironment of discs in vivo. Certainly this in vivo differentiate research will continue to provide a powerful tool to investigate the biological behavior and function of WJCs.

A number of studies have shown that degradative enzymes were highly produced by NP cells in degenerate IVDs [59, 60]. Matrix metalloproteinase (MMP) gene expression, however, could be downregulated in NP cells after coculture with MSCs [51]. Another possible mechanism of delaying IVD degeneration might therefore be that intradiscal transplantation of WJCs affected the endogenous NP cells by inhibiting the expressions of MMP genes or upregulating the expressions of SOX-9 and ECM genes, as shown by the results of genetic evaluation in the present study.

Some species such as rabbits [50], rats [58], pigs [53], and beagles [44] have been used as animal models for cell transplantation studying in IVD degeneration [61, 62]. In this study, beagles were used as an animal model because the beagle model of disc degeneration was closer to human morphology and was of the same status in human [44, 63]. To eliminate individual differences such as weight, infection status, activity level, and immune competence, we chose the experimental model in which control and experimental segments were present in the same animal. We fixed assignment of discs to a specific group, and we believed that lumbar biomechanical stress might not have significant differences from level to level owing to the fact that beagles couldn't walk upright. However, randomization of the disc levels would be the best study design. The discs of beagles are prone to degeneration at an earlier age, which might affect individual IVDs to a different extent. Thus, there might be potential bias in the study. The beagle was a chondrodystrophic breed, and the NP of young beagle consisted mainly of notochordal cells and was gradually replaced by chondrocyte-like cells with aging. Gillett et al. [64] showed that the NP of 2-year-old beagles had vacuolar degenerative notochordal cells and cloning of cartilage in the transitional zone (TZ). Consistent with the study, we also found vacuolar degenerative notochordal cells in the NP and cloning of cartilage in the TZ in 1.5-year-old beagles. Moreover, the degeneration of discs in the CS and TS groups was slightly higher than that of discs in the DS and FS groups.

There are many methods including mechanical compression, mechanical instability, structural injury, and chemical injury to establish an animal model of IVD degeneration [65]. In this study, partial aspiration of the NP was used to establish an animal model of degeneration. However, there were shortcomings to this method, because this IVD degeneration model might not truly represent human disc degeneration. In human IVD, the balance between matrix synthesis and degradation was destructed with aging which induced progressive degeneration and pathologic alteration of IVD, and this was related to the bad microenvironment of low pH, reduction in nutrients, and increased inflammatory cytokines. In this animal model, however, the IVD degeneration was caused by aspiration of NP tissues, which decreased the number of NP cells and resulted in decrease in proteoglycan content and disc height. Although this was a shortcoming of this model, there was no suitable animal model that could completely mimic human IVD degeneration up to now [56]. For this reason, we chose the aspiration-induced model of degeneration in this study. Moreover, this model had been used in many cell transplantation studies [9, 44, 56, 57, 66]. In our study, the NP of the DS group was observed to undergo degeneration by the gradual decrease of %DHI and RGI from the results of radiological and MRI analysis, so the degeneration was successfully induced in this animal model.

Many studies have reported that the transplantation of MSCs could decelerate the process of disc degeneration in an animal model [44]. The safety and effectiveness of BMSC and WJC transplantation have been confirmed in clinical research [67, 68]. What are the advantages of using WJCs in the clinical setting compared with adult MSCs? First, WJCs express HLA class I and do not express HLA class II surface markers, and are immune suppressive in mixed lymphocyte assays and inhibit T-cell proliferation [69–73]. Moreover, like other adult MSCs, WJCs are immunologically privileged, do not require tissue matching, and can be tolerated in allogeneic and xenogeneic transplantation [32] because any donor can supply the cells to any other patient without need for immunosuppressant drugs [70]. Second, WJCs are perinatal stem cell sources which represent a bridge between embryonic and adult stem cells, and have great potential to serve as a useful stem cell source to treat various diseases in the clinics [74]. Third, the umbilical cord is abundant and easy to obtain. Use of the human umbilical cord tissues involves mild ethical and less religious controversies [31, 75]. This source of stem cells allows the rapid initial isolation of large numbers of cells, avoiding the necessity of extensive multiplication and potential epigenetic damage [76]. Fourth, compared with other adult MSCs, WJCs have shorter doubling times, and greater ex-vivo expansion capabilities and numbers of passages to senescence [77]. Finally, cells derived from Wharton’s jelly have a higher frequency of colony-forming unit fibroblasts as compared with that of BMSCs [78, 79]. Taken together, these studies suggested that the WJCs could serve as a valuable cell resource instead of other original MSCs. The results of our study also suggest that WJCs might be a better candidate for a cell-based treatment for degenerative disc disease in clinical application.

## Conclusions

Our study demonstrated that WJC transplantation maintained the disc height and T2-weighted signal intensity, and promoted the disc matrix formation of aggrecan and type II collagen in degenerate IVDs. WJCs are a potential cell source for disc regeneration and have important value in the treatment of degenerative disc disease.

## Abbreviations

AAV2: Adeno-associated virus 2
ADSC: Adipose-derived stem cell
AF: Annulus fibrosus
BMSC: Bone marrow mesenchymal stem cell
Ct: Cycle threshold
DHI: Disc height index
DMEM: Dulbecco's modified Eagle’s medium
ECM: Extracellular matrix
EGFP: Enhanced green fluorescent protein
FBS: Fetal bovine serum
FITC: Fluorescein isothiocyanate
GFP: Green fluorescent protein
HLA: Human leukocyte antigen
HNA: Mouse antihuman nuclei
IVD: Intervertebral disc
LBP: Low back pain
MMP: Matrix metalloproteinase
MRI: Magnetic resonance imaging
MSC: Mesenchymal stem cell
NP: Nucleus pulposus
PBS: Phosphate-buffered saline
PE: Phycoerythrin
RGI: Relative gray index
ROM: Range of motion
SOX-9: SRY-box 9
TZ: Transitional zone
WJC: Wharton’s jelly cell
